# Evaluation and Feedback for Telehealth From Patients and Physicians During the Early Stage of COVID-19 Pandemic Period

**DOI:** 10.7759/cureus.12633

**Published:** 2021-01-11

**Authors:** James Yu, Summia Matin Afridi, Ashley C Cozart, Luis Isea, Jian Guan

**Affiliations:** 1 Internal Medicine, AdventHealth Orlando, Orlando, USA; 2 Internal Medicine, Bassett Medical Center, Cooperstown, USA; 3 Internal Medicine, University of Central Florida, Orlando, USA

**Keywords:** covid19 pandemic, evaluation for telehealth, patients and physicians

## Abstract

Purpose

Many health care providers adopted telehealth during the coronavirus disease 2019 (COVID-19) pandemic. This unprecedented transformation in medical practice posed challenges to both physicians and patients. However, little is known about the adaptation of attendings, residents, and patients to this new normal. Thus, a survey was sent out to investigate the feedback of both physicians and patients on telehealth.

Methods

Surveys were administered via phone call to patients and electronic survey to physicians at an internal medicine resident clinic in one tertiary community hospital from April to June 2020. Demographic information and assessment of overall experience, satisfaction, and concerns of telehealth were collected. Statistical analyses were performed to compare feedback between patients and physicians.

Results

Fifty patients and 45 physicians participated in the study. Eighty-four percent of patients were first- or second-time users, and 50% of patients were older than 60 years. Eighty-four percent of patients were very or extremely satisfied with telehealth, while 72% wanted to continue telehealth in the future. Ninety-four percent of patients believed that their concerns were adequately addressed, but 14% experiencing technical issues. Physicians' feedback to telehealth was less positive than the patients'. More than 60% of physicians experienced technical issues, and nearly 60% of physicians were neutral or not satisfied with telehealth. Nearly 50% of physicians had difficulty transitioning to telehealth, while only 29% believed that their patients’ complaints were adequately addressed. Most physicians had to schedule in-person visits after telehealth. Patients were more satisfied with telehealth than physicians (84% vs. 42%; p<0.001) and were more likely to believe that their concerns were properly addressed by telehealth (94% vs. 29%; p<0.001).

Conclusion

This survey revealed that patients were more satisfied with telehealth than physicians. Further research with a larger sample should be considered to confirm this conclusion, and subjective studies are needed to determine the imbalance of satisfaction.

## Introduction

Coronavirus disease 2019 (COVID-19) cases are increasing significantly in the United States. As of September 13th, 2020, there are around 6.6 million total cases with 196,000 deaths [1]. Based on the suspicion that COVID-19 is a respiratory infectious disease, social distancing has been a key component of disease control since the beginning of the outbreak [2]. As a result, the necessity of telehealth, or the use of medical services remotely through telecommunication technology, in healthcare has accelerated drastically [3].

Although some telehealth technologies have existed for decades, telehealth has rarely adapted into patient care due to heavy regulation, lack of infrastructure, and lack of known cost-effectiveness [4, 5]. For example, in a 2019 PwC Health Research Institute survey, 38% of chief executive officers of the U.S. health care systems reported having no digital component in their overall strategic plan, mainly due to data-protection and Health Insurance Portability and Accountability Act (HIPAA) issues [6].

However, to cope with the COVID-19 pandemic, most healthcare providers have developed telehealth models into their practice. The government has also encouraged this practice. For example, on April 2nd, 2020, the Federal Communications Commission (FCC) established a $200 million fund for “COVID-19 for Telehealth Program” to help healthcare providers provide connected care services to patients in response to the pandemic [7].

Telehealth can provide remote assessment and care for COVID-19 confirmed and suspicious patients. Likewise, it can provide convenient and safe access to routine care for patients that are not infected with the COVID-19, especially those at higher risk of infection, such as elderly patients with pre-existing comorbidities. Telehealth also reduces patient exposure to health care providers by allowing for social distancing.

Although telehealth has many benefits in the COVID-19 era, this unprecedented transformation in medical practice poses great challenges to both physicians and patients. Little is known about the adaptation of physicians and patients to this new normal. Thus, a survey was created to investigate the feedback of both physicians and patients on telehealth. Multi-aspect issues regarding telehealth were analyzed, including the difficulty of transition, patient satisfaction, and its effect on the physician-patient relationship.

## Materials and methods

Patients who experienced telehealth visits at least once in a single institution, internal medicine, or family medicine clinic were eligible for this survey. Anonymous surveys with multiple questionnaires were conducted via phone call to patients or electronic survey to faculty and residents via SurveyMonkey® platform (SVMK Inc., San Mateo, CA) from April 7th to June 25th, 2020.

All physicians were provided instructions on how to provide virtual video visits. During these visits, the patient communicated with a physician via video call and described his or her symptoms. The physical exam was limited due to the nature of the video call. The patient’s medications and labs were sent electronically to the pharmacy and laboratory, respectively. Visits were conducted via Doximity (Doximity, San Francisco, CA) or Doxy.me (Doxy.me, LLC, Rochester, NY) platform. Patients were provided instructions on how to use these platforms by the nursing staff before the start of their visit with the physicians, and consent was obtained.

Patient survey

A six-question cross-sectional survey for the patients was conducted over the phone. Answers were anonymously recorded. Patients that required language interpreter services were excluded from the survey. Patients were called within 48 hours of their telehealth visit by a team of residents to complete the survey over the phone. Residents conducting the survey were not a part of the patient’s treatment team. Questions assessed patients’ previous experience with telehealth visits, ease of use, technical difficulties, and overall satisfaction using a Likert scale ranging from 1 (not at all satisfied) to 5 (extremely satisfied). Patients were asked if they believed that their medical concerns were adequately addressed during the telehealth visit compared to an in-person visit and the likelihood that they would continue using telehealth visits after the pandemic.

Physician survey

A ten-question survey was designed administered to physicians by the online SurveyMonkey® platform. Position - resident (postgraduate year [PGY] 1, 2 or 3) versus attending physician, satisfaction with telehealth visits, technical difficulties, number of in-person visits scheduled after telehealth visit, and number of times used. Difficulty in transitioning to telehealth from in-person visits and likelihood to continue telehealth in the future were also evaluated. Physicians were also asked if they preferred telephone visits over video visits, and if they think that the patient’s concerns were adequately addressed during the telehealth visit. Physicians were also asked about concern for compromise of the patient-physician relationship during telehealth visits compared to in-person visits. Questions included in both patient and physician surveys included satisfaction, ease of use, technical difficulties, willingness to continue using telehealth after the pandemic, and whether concerns were adequately addressed.

Statistical analyses

Statistical analyses were performed by using Fischer’s exact test to compare the rate of satisfaction, and concerns were adequately addressed between patients and physicians. Responses of “very satisfied” or “extremely satisfied” to the question "How satisfied are you with telehealth visits?" were classified as satisfactory. Responses of “yes” to the question “Do you think that patients’ complaints are adequately addressed during telehealth visits?” were compared between each group. The type I error rate was fixed at 0.05. Statistical analyses were performed with STATA software version 15.1 (StataCorp, College Station, TX).

## Results

A total of 50 patients and 45 physicians participated in our survey.

Patients’ answers

Table 1 summarized patients’ responses. Most (84%; 42/50) of patients in our cohort were first- or second-time telehealth users, 50% of patients were older than 60 years, and 60% were female. The majority of patients had positive experiences regarding telehealth visits. Eighty-four percent (42/50) of patients were very or extremely satisfied with the telehealth visits, and 72% (36/50) are likely to continue telehealth visits after completing social distancing from COVID-19. Ninety-four percent of patients felt their concerns were adequately addressed, but 14% experienced technical issues during visits.

**Table 1:** Summary of patient survey responses

Patient characteristics (n=50)	Feedback	# of respondents
Age (year)	<40	8 (16%)
	40--59	17 (34%)
	>60	25 (50%)
Gender	Female	30 (60%)
	Male	20 (40%)
Total telehealth visits	1 time	34 (68%)
	2 times	8 (16%)
	3 times or more	8 (16%)
Difficulty to use telehealth (0 being the easiest and 5 being the most difficult)	0 - 1	38 (76%)
	2 - 3	6 (12%)
	4 - 5	6 (12%)

Physicians’ answers

Table 2 summarized patients’ responses. Our physician cohort consisted of PGY1, PGY2, PGY3 residents, and attending physicians, and represented 31%, 22%, 20%, and 27%, respectively. Fifty-one percent (23/45) of physicians experienced telehealth encounters between 1-5 times, while 49% experienced more than six times. In contrast to patients, physician feedback was less positive than patients. Fifty-eight percent (26/45) of physicians were neutral or not satisfied with telehealth, and nearly half of physicians worried about the physician-patient relationship being compromised due to virtual visits. More than 60% of physicians experienced technical issues during encounters, and only 29% of physicians felt the patients’ complaints were adequately addressed. Half of the physicians answered they had to schedule in-person visits after video visits. More physicians answered they prefer video (58%) visits to telephone (22%).

**Table 2:** Summary of physician survey responses PGY - postgraduate year

Physician characteristics (n=45)	Feedback	# of respondents
Residency positions (n=45)	PGY1	14 (31%)
	PGY2	10 (22%)
	PGY3	9 (20%)
	Attending	12 (27%)
Number of telehealth encounters	1-5 times	23 (51%)
	6-10 times	7 (16%)
	11 times or more	15 (33%)
Difficulty to transition to telehealth (0 being the easiest and 5 being the most difficult)	0 - 1	22 (49%)
	2 - 3	17 (38%)
	4 - 5	6 (13%)
Do you prefer video visits over telephone visits?	Yes	26 (58%)
	No	9 (20%)
	No preference	10 (22%)
Are you worried about the physician-patient relationship being compromised due to virtual visits?	Yes	12 (27%)
	No	24 (53%)
	Somewhat	9 (20%)
Did you have to schedule a patient for an in-person visit after a video visit?	Never	8 (18%)
	Rarely	14 (31%)
	Sometimes	22 (49%)
	Often	1 (2%)
	Always	0 (0%)

Comparisons between patients and physicians

**Figure 1:**
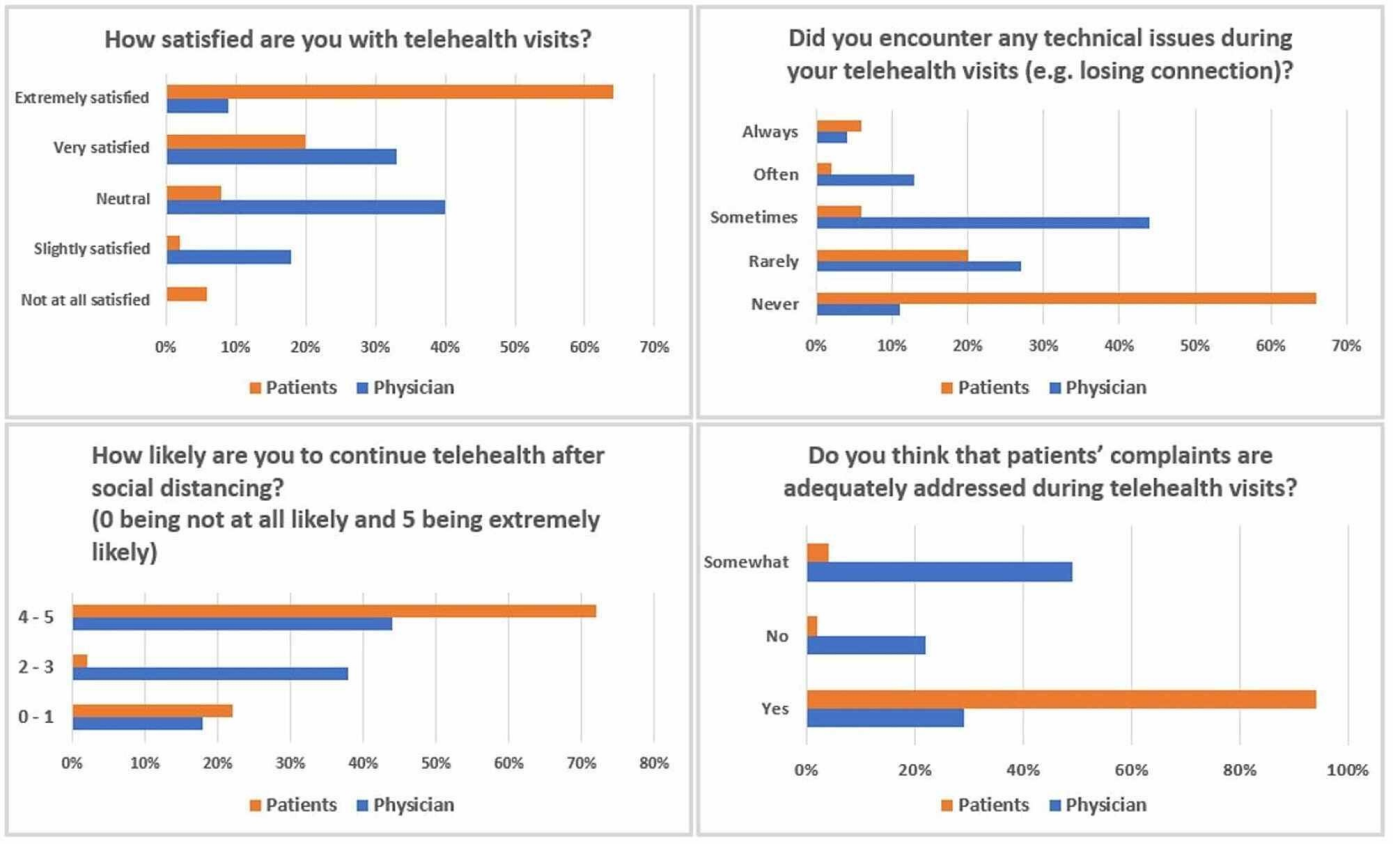
Comparison between patients and physicians

Interestingly, in our statistical analyses, there was a statistically significant difference in the satisfaction rate between patients and physicians, with patients being more satisfied with their visits (84% vs. 42%; p<0.001). Also, 94% of patients thought that their concerns were properly addressed, while only 29% of physicians thought that the patient's concerns were properly addressed (p<0.001). Table 3 and Figure 1 show comparisons between patients and physicians in our survey.

**Table 3:** Comparison of responses between patients and physicians

Survey item	Feedback	# of respondents	
		Physician (n=45)	Patients (n=50)
How satisfied are you with telehealth visits?	Not at all satisfied	0 (0%)	3 (6%)
	Slightly satisfied	8 (18%)	1 (2%)
	Neutral	18 (40%)	4 (8%)
	Very satisfied	15 (33%)	10 (20%)
	Extremely satisfied	4 (9%)	32 (64%)
How likely are you to continue telehealth after social distancing? (0 being not at all likely and 5 being extremely likely)	0 - 1	8 (18%)	11 (22%)
	2 - 3	17 (38%)	3 (2%)
	4 - 5	20 (44%)	36 (72%)
Do you think that patients’ complaints are adequately addressed during telehealth visits?	Yes	13 (29%)	47 (94%)
	No	10 (22%)	1 (2%)
	Somewhat	22 (49%)	2 (4%)
Did you encounter any technical issues during an individual or in multiple telehealth visits (e.g. losing connection)?	Never	5 (11%)	33 (66%)
	Rarely	12 (27%)	10 (20%)
	Sometimes	20 (44%)	3 (6%)
	Often	6 (13%)	1 (2%)
	Always	2 (4%)	3 (6%)

## Discussion

Many health care providers have adopted the telehealth model to comply with social distancing and minimize unnecessary exposure during the COVID-19 pandemic [8]. This survey gleaned insight into the adaptation and satisfaction of internal medicine residents, faculty, and patients to the new practice of telehealth. Patients in the current study were more satisfied and more likely to continue telehealth than physicians. There are conflicting data on patient satisfaction with telehealth in the literature. Some studies have reported resistance to change [9, 10], specifically in the elderly population [11], while others have reported acceptance and embrace to change [12-14]. The patients in our sample support the latter. Although specific reasons for the satisfaction were not obtained in our study, there are many possible explanations for this phenomenon. In the systematic review about telehealth by Kruse and colleagues, ease of use, improved communication, low cost, decreased travel time, more empowerment to manage chronic conditions, decreased missed appointments, good modality for education, and decreased wait times were included as main reasons for patients’ high satisfaction for telehealth [14]. Additional explanations during the COVID-19 era include decreased patient anxiety about virus exposure.

Physicians, on the other hand, were not as satisfied as patients in our sample. While 94% of patients thought that their concerns were adequately addressed, only 29% of physicians thought similarly. Also, about half of the physicians felt like they needed to schedule in-person visits after the telehealth visit. This low satisfaction contrasts with prior studies on physician satisfaction [15, 16]. Our results reveal that physicians could be worried about compromising the physician-patient relationship due to a lack of in-person communication. Other factors could also play a role, including a perceived lack of ease of use of telehealth service [17], because many physicians experienced technical issues throughout telehealth visits. A prior study by Kissi and colleagues revealed positive links between physicians’ satisfaction of telehealth services with perceived ease of use, perceived usefulness, behavioral intention, and attitude of use [17]. Our results support the need to incorporate these determinants into the telehealth practice.

Additionally, data in the current study was obtained at an internal medicine residency clinic, with both resident and attending physician responses comprising the “physician” data. This could contribute to low physician satisfaction, as providing telehealth services during training came as a surprise to many residents. Many residents at this clinic had no prior exposure to telehealth. A study by Kirkland and colleagues found that incorporating a three-year longitudinal telehealth curriculum for internal medicine resident physicians drastically improved resident comfort and ability to provide telehealth services [18]. This study and the low physician satisfaction with telehealth reveal the potential need to incorporate a telehealth curriculum into internal medicine resident training, especially in the COVID-19 era.

Further, there were significant technical issues reported from both patients and physicians in our sample. In our sample, technology was a barrier to successfully implementing telehealth models as evidenced by physician satisfaction, which is consistent with prior studies [17, 19, 20]. This demonstrates the need to focus on both technical and process improvement when implementing telehealth models into practice.

This survey-based study has several limitations. First and foremost, as with any survey study, there is a potential for response bias, in which participants could respond inaccurately to questions. Also, the responses were obtained from an internal medicine resident clinic, specifically with a relatively small sample size, which could decrease the results' generalizability. Further research with physicians and patients from different medical departments with a larger population is warranted to confirm the result. Additionally, the current study only requested quantitative data, including forced responses to survey questions, without assessing qualitative data such as subjective reasoning for dissatisfaction or identifying the main technical issues that patients or physicians experienced. To better understand the decreased physician satisfaction with telehealth or the reason physicians experienced more technical issues compared to patients, further research focusing on more subjective data, such as in-depth interviews, is warranted.

## Conclusions

As the COVID-19 pandemic is prolonging, the transformation to telehealth in medical practice is expected to remain for quite some time. We performed a survey-based analysis to investigate the evaluation and satisfaction of physicians and patients to telehealth visits at an internal medicine resident clinic. Patients in our sample showed higher overall satisfaction to telehealth than physicians. Further research with a larger sample should be considered to confirm this conclusion, and further subjective studies are needed to determine the imbalance of satisfaction.
